# Veterinary medicine under COVID-19: a mixed-methods analysis of student and practitioner experiences in Austria

**DOI:** 10.3389/fvets.2024.1460269

**Published:** 2024-11-06

**Authors:** Elke Humer, Stefanie Winter, Thomas Probst, Christoph Pieh, Rachel Dale, Deianira Brühl, Viktoria Neubauer

**Affiliations:** ^1^Department for Psychosomatic Medicine and Psychotherapy, University for Continuing Education Krems, Krems an der Donau, Austria; ^2^Faculty of Psychotherapy Science, Sigmund Freud University Vienna, Vienna, Austria; ^3^Division of Psychotherapy, Department of Psychology, University of Salzburg, Salzburg, Austria; ^4^Centre for Food Science and Veterinary Public Health, Clinical Department for Farm Animals and Food System Science, University of Veterinary Medicine Vienna, Vienna, Austria; ^5^FFoQSI GmbH - Austrian Competence Centre for Feed and Food Quality, Safety and Innovation, Tulln, Austria

**Keywords:** COVID-19, pandemic, veterinarians, veterinary students, Austria, corona virus, mental health

## Abstract

**Background:**

This study aimed to provide a detailed analysis of the pandemic’s impact on the veterinary profession and education in Austria.

**Methods:**

Two online surveys were conducted from November 2022 to January 2023, inviting all veterinarians and veterinary students in Austria to share their experiences on the impact of the pandemic on their veterinarian work and education in a free text question.

**Results:**

A total of *n* = 289 veterinarians and *n* = 272 veterinary students provided an answer. In summary, 39.8% of veterinarians reported no impact by the pandemic, while others experienced changes in workload (19.7%), changes in client behavior (14.9%), implementation of safety measures (13.1%), and organizational changes (13.1%). Additional impacts included effects on mental health, private life, finances, as well as physical health. Changes were more frequently mentioned in veterinarians working with pets compared to those working with livestock. In the student panel, only a minority reported no significant impact (8.1%) but 44.9% reporting changes due to online and hybrid learning. Key issues included impaired learning conditions (34.9%), and social distancing (34.6%) leading to social isolation. Some students appreciated increased flexibility and reduced commuting (10.7%), while others experienced mental health challenges (10.7%). Further impacts were related to the implementation of safety measures (5.5%), organizational changes (4.8%) and impaired physical health due to infection (1.1%). Negative changes related to distance learning were mainly reported by students in the final study phase, whereas those in the first study phase reported more positive aspects related to online/hybrid learning.

**Conclusion:**

Overall, this study highlights the significant impact of the COVID-19 pandemic on veterinary education and professional practice in Austria, revealing diverse challenges for students and relatively lower but still notable effects on practicing veterinarians. Future research should monitor these impacts longitudinally and explore the integration of beneficial practices into standard veterinary education and care, such as effective digital learning platforms and appointment-based systems.

## 1 Introduction

The COVID-19 pandemic has significantly disrupted various aspects of life worldwide, including the fields of veterinary medicine and education. SARS-CoV-2, the etiological agent of COVID-19, is both zoonotic and anthropozoonotic, meaning it can be transmitted between animals and humans, highlighting the interconnectedness of human and animal health (1, 2). While the virus is believed to have originated from a zoonotic source, its spillover into humans and subsequent anthropozoonotic transmission to other species has raised concerns, particularly regarding companion animals such as dogs and cats. Research has shown that cats, in particular, exhibit greater susceptibility to SARS-CoV-2 compared to dogs. However, no severe clinical symptoms have been reported in pets (3, 4) and animal-to-human transmissions are considered negligible (3, 5).Globally, the pandemic has had profound effects on healthcare professionals, with numerous studies highlighting increased stress, burnout, and mental health issues among frontline workers (6–8). Similar trends have been observed in the veterinary field, where professionals have reported increased workload, changes in client behavior, and the need for enhanced safety protocols (9, 10). For instance, previous studies reported heightened mental distress due to increased demand for animal healthcare, changes in client interactions, the implementation of new safety measures and the fear of infection (11–13).

Worldwide, students also experienced significant disruptions. In several countries, education institutions were required to shift their teaching and assessment activities to novel modes of content delivery and assessment schemes (14, 15). Research has shown that the transition to online learning often posed substantial challenges, particularly in fields that rely heavily on practical, hands-on training (16–18). The loss of in-person classes, labs, and clinical rotations not only affected medical students’ academic performance, but also their mental health, with many reporting increased stress, anxiety, depression and decreased motivation (17, 19, 20). However, there seem to be large variations among universities and online modalities. As an example, virtual clinical rotations at a veterinary medicine college in the US were evaluated equivalent or even better by students compared to the corresponding in-person rotations of the previous year (21).

In Austria, veterinarians and veterinary students have faced unique challenges as they adapted to the rapidly changing circumstances caused by the pandemic and the associated containment strategies of the government and teaching facilities. We recently reported higher prevalence rates of mental health symptoms in Austrian veterinary students as well as veterinarians compared to the general public at the end of the COVID-19 pandemic (22, 23). However, there is a lack of information on the specific impacts of the pandemic on veterinary professionals and students in Austria, focusing on how these changes have affected their professional and educational experiences.

In Austria, the COVID-19 pandemic began with the first confirmed cases in late February 2020 (24). Like many other countries, the Austrian government implemented strict measures to curb the spread of the virus. These measures included lockdowns, social distancing, mandatory mask-wearing, and restrictions on gatherings (25). Specific measures for practicing veterinarians included: staying at home if showing signs of illness, implementing appointment-based consultations, minimizing customer contact and examination time (wherever feasible), maintaining physical distance (at least one to two meters), mandatory face masks for patients visiting clinics, mandatory face masks for staff with customer contact, strongly recommending FFP2 masks (along with face shields or goggles and gloves for veterinarians, assistants, and staff), installing (plexiglass) barriers at reception, avoiding handshakes and not touching the face, thorough handwashing several times a day for at least 30 s, and hand disinfection after each patient or customer contact (26). Educational institutions, including universities, were forced to switch to online learning platforms almost overnight (27). Non-essential businesses were closed, causing a sharp rise in unemployment. Essential services had to operate under new and often restrictive guidelines. Despite these challenges, veterinary services were classified as essential, allowing veterinarians to continue their work under modified conditions. By late March 2020, daily confirmed cases peaked, and active cases reached their highest in early April (24). From mid-April, restrictions were gradually eased, allowing some normality to return, though economic recovery was slow (28). During the summer of 2020, regional clusters and travel returnees led to renewed measures like mask mandates and increased testing. A second wave of COVID-19 cases in autumn 2020 prompted further restrictions and the introduction of monitoring tools. In November 2020, a renewed lockdown began, lasting through early 2021 with intermittent easing (29). A vaccination campaign started in January 2021 despite supply shortages, and mass testing and mask mandates were introduced (30). In early 2021, a third wave of infections brought regional lockdowns. By late May 2021, vaccination rates improved significantly, aided by increased testing availability (31). Summer 2021 saw eased measures due to declining infections and the rollout of the “Green Passport” and “3G” rule. However, the spread of the Delta variant in late 2021 led to new restrictions and a national lockdown in November, with a vaccine mandate announced for February 2022 (32). The general lockdown ended in December 2021, though a “lockdown for the unvaccinated” continued until January 2022. The Omicron variant led to further challenges due to new highs in confirmed COVID-19 cases, but with milder disease courses, many restrictions were lifted by spring 2022 (33). By summer 2022, most COVID-19 measures were withdrawn, including the mask mandate and vaccine mandate, despite a summer wave of infections (34). At the end of the third year of the pandemic (winter 2022/2023), restrictions were minimal, limited to mask mandates in healthcare settings and mobility restrictions for individuals infected with COVID-19.

The impacts of the COVID-19 pandemic and associated containment efforts on veterinarians and veterinary students in Austria have not been assessed so far. This study seeks to fill the gap in understanding the specific impacts of the COVID-19 pandemic on veterinarians and veterinary students in Austria. By examining both the professional and educational challenges faced by these groups, this research aims to provide a comprehensive overview of how the pandemic has affected their work and studies.

This study addressed the following research questions:

1. What were the immediate and indirect impacts of the COVID-19 pandemic and its associated containment strategies on the professional work of veterinarians in Austria?
2. Are there any differences in the reported impacts of the COVID-19 pandemic including containment measures on the work of veterinarians in Austria with respect to gender, age, professional field, treated species, and employment status?
3. How did the COVID-19 pandemic and the strategies to combat the spreading of the virus affect the educational experience of veterinary students in Austria?
4. Are there any differences in the reported impacts of the COVID-19 pandemic including the strategies implemented to contain the pandemic on the veterinary education in Austria with respect to gender, country of origin, and study phase?

## 2 Materials and methods

### 2.1 Study design

Between November 16, 2022, and January 31, 2023, two cross-sectional online surveys were conducted targeting Austrian veterinary students and licensed Austrian veterinarians. The surveys were part of a larger study that assessed the mental health status of veterinarians and veterinary medicine students, as well as profession-specific stressors (22, 23, 35). These surveys also aimed to explore the impact of the COVID-19 pandemic on their professional and academic lives. All students enrolled in the veterinary medicine diploma program in Austria (*N* = 1,477) were invited to participate through notifications sent by the Union of Students of the University of Veterinary Medicine Vienna and the university’s registrar’s office. Additionally, invitations were emailed to all registered veterinarians listed with the Austrian Chamber of Veterinarians who had provided valid email addresses (*N* = 4,534). The surveys were conducted using the LimeSurvey platform (LimeSurvey GmbH, Hamburg, Germany). Participation was voluntary and no incentives were provided.

### 2.2 Ethical considerations

The study adhered to the ethical principles outlined in the Declaration of Helsinki and received approval from the Ethics Committee of the University for Continuing Education Krems, Austria (Ethical number: EK GZ 25/2021–2024). All participants provided electronic informed consent prior to completing the questionnaires.

### 2.3 Measures

#### 2.3.1 Sociodemographic characteristics

Students and practicing veterinarians were asked about their age and gender. Students were further asked about their country of origin (Austria or other countries).

#### 2.3.2 Professional and study-related characteristics

Veterinarians were asked about their years in the profession, the animal species they worked with, which included ruminants, pigs, horses, poultry, pets, and exotic animals. Information on their employment status (employed, self-employed, both) and professional field [curative practice, non-curative (university/research, consulting, abattoir, animal and meat inspection, official veterinarian)] was also collected. For further analyses, ruminants, pigs, and poultry were summarized within the category “livestock,” all other fields than curative practice were summarized as “others” and the employment status was dichotomized into those veterinarians being self-employed or self-employed and employed vs. those being not self-employed.

Students were asked to specify their current semester of study. The study phase was categorized according to the curriculum of the veterinary medicine diploma program as follows: Phase 1 (fundamental knowledge about living organisms, 1st–4th semester), Phase 2 (advanced understanding of physiological and pathological processes, 5th–9th semester), and Phase 3 (specialization, 10th–12th semester).

#### 2.3.3 Impact of the pandemic

Veterinarians were asked the open-ended question: “What direct or indirect impacts has the pandemic had on your work as a veterinarian?” Similarly, students were asked: “What direct or indirect impacts has the pandemic had on your veterinary medicine studies?” No time frame for the pandemic was specified, allowing participants to interpret the period based on their own experiences. The inclusion of this open-ended questions allowed participants the opportunity to provide detailed responses. However, it was also possible for them to continue the questionnaire without answering these questions.

### 2.4 Qualitative data analysis

The qualitative data were analyzed using a traditional content analysis approach (36), following an iterative process for qualitative data analysis (37). Initially, one coder carefully read through all responses in the sample, which ranged from single words to lengthy expressions. During this reading, an initial list of inductive categories was developed. These categories were then thematically sorted and organized into higher-level main categories. The preliminary category system was iteratively discussed and refined by the research team. Thereafter, the entire dataset was coded using this system, adding new inductive categories as needed. Any ambiguous text passages were discussed with the research team. After completing the coding, the category system was reviewed to ensure distinctiveness, appropriate subcategory assignments, and to consolidate smaller categories into broader, more abstract ones.

### 2.5 Mixed-methods analysis

Following the qualitative analysis, a mixed-methods approach was employed to examine differences based on sociodemographic, professional (veterinarians) and study-related (students) variables. This analysis was conducted using SPSS version 26 (IBM Corp, Armonk, NY, USA). Chi-squared tests (*χ*^2^) or Fisher’s exact tests were utilized to investigate differences within main or subcategories of reported answers for:

- Gender, age (categorized into 24–34 years, 35–44 years, 45–54 years, ≥55 years), professional field (curative practice vs. non-curative practice), employment status (self-employed vs. employed) and treated animal species (pets, livestock, horses, exotic animals) in veterinarians.
- Gender, country of origin (Austria vs. abroad), and study phase in students.

Due to the low number of gender-diverse individuals, only male and female participants were considered in these analyses.

All tests were two-tailed, with a significance level set at 5%.

## 3 Results

### 3.1 Study sample characteristics

A total of *n* = 608 veterinarians clicked on the survey link, of whose *n* = 440 (72.4%) completed the entire survey and detailed information on their sociodemographic and professional characteristics are presented elsewhere (23). From those veterinarians who completed the survey, a total of *n* = 289 veterinarians (65.7%) provided an answer to the open-ended question on the impact of the pandemic on their professional activities.

Veterinarians who answered the question were older (46.14 ± 11.20 years vs. 41.44 ± 10.72 years; *p* < 0.001), more years in the profession (18.29 ± 10.83 years vs. 13.98 ± 10.08 years; *p* < 0.001), and more frequently self-employed (57.1% vs. 47.7%; *p* = 0.009), but did not differ with respect to gender, main professional field and treated animal species to those who did not respond to the question (Table 1).

**Table 1:** Veterinarians study sample characteristics (*N* = 440).

Variable	Responders (*N* = 289)	Non-responders (*N* = 151)	Statistics
Gender			
Female, % (*N*)	69.6 (201)	76.8 (116)	*χ*^2^ (1) = 2.60; *p* = 0.107
Male, % (*N*)	30.4 (88)	23.2 (35)	
Age in years, M (SD)	46.14 (11.20)	41.44 (10.72)	*t* (316.2) = − 4.24; *p* < 0.001
Years in the profession, M (SD)	18.29 (10.83)	13.98 (10.08)	*t* (324.0) = − 4.15; *p* < 0.001
Employment status			
Employed, % (*N*)	36.0 (104)	49.7 (75)	*χ*^2^ (2) = 9.52;*p* = 0.009
Self-employed, % (*N*)	57.1 (165)	47.7 (72)	
Both, % (*N*)	6.9 (20)	2.6 (4)	
Professional field			
Curative practice, % (*N*)	87.2 (252)	89.4 (135)	*χ*^2^ (1) = 0.46;*p* = 0.500
Others, % (*N*)	12.8 (37)	10.6 (16)	
Species			
Livestock, % (*N*)	38.4 (111)	36.4 (55)	*χ*^2^ (1) = 0.17; *p* = 0.683*χ^2^* (1) = 0.23; *p* = 0.636*χ*^2^ (1) = 3.04; *p* = 0.081*χ*^2^ (1) = 0.03; *p* = 0.855
Horses, % (*N*)	38.1 (110)	35.8 (54)	
Pets, % (*N*)	74.0 (214)	81.5 (123)	
Exotic animals, % (*N*)	15.2 (44)	14.6 (22)	

A total of *n* = 430 students participated in the survey. Completion rate was 79.5% (430 out of 541 students who clicked on the survey link completed the survey). Characteristics of this sample are reported in detail in our companion study (22). A total of *n* = 272 students (63.3%) provided an answer to the open-ended question on the impact of the pandemic on their studies. Compared to non-responders, students who answered the question were more frequently in a later study phase (*p* < 0.001), but did not differ with respect to age, gender, and country (Table 2).

**Table 2:** Veterinary students study sample characteristics (*N* = 430).

Variable	Responders (*N* = 272)	Non-responders (*N* = 158)	Statistics
Gender			
Female, % (*N*)	87.1 (237)	83.5 (132)	χ^2^ (2) = 4.92; *p* = 0.064
Male, % (*N*)	12.9 (35)	14.6 (23)	
Diverse, % (*N*)	0 (0)	1.9 (3)	
Age in years, M (SD)	23.39 (3.56)	22.72 (3.88)	*t* (428) = − 1.81; *p* = 0.070
Semester, M (SD)	6.24 (3.64)	4.89 (3.61)	*t* (428) = − 3.73; *p* < 0.001
Country of origin			
Austria, % (*N*)	70.6 (192)	61.4 (97)	*χ^2^* (1) = 3.84; *p* = 0.050
Other countries, % (*N*)	29.4 (80)	38.6 (61)	

The *n* = 141 students from other countries included individuals from Germany (*n* = 100), Italy (*n* = 19), Luxembourg (*n* = 5), Hungary (*n* = 4), Belarus (*n* = 2), Poland (*n* = 2), Serbia (*n* = 2), Slovakia (*n* = 2), and Iran (*n* = 1), while the remaining *n* = 4 students did not provide information on their country of origin.

### 3.2 Impact of the pandemic on the work of veterinarians

**Figure 1:**
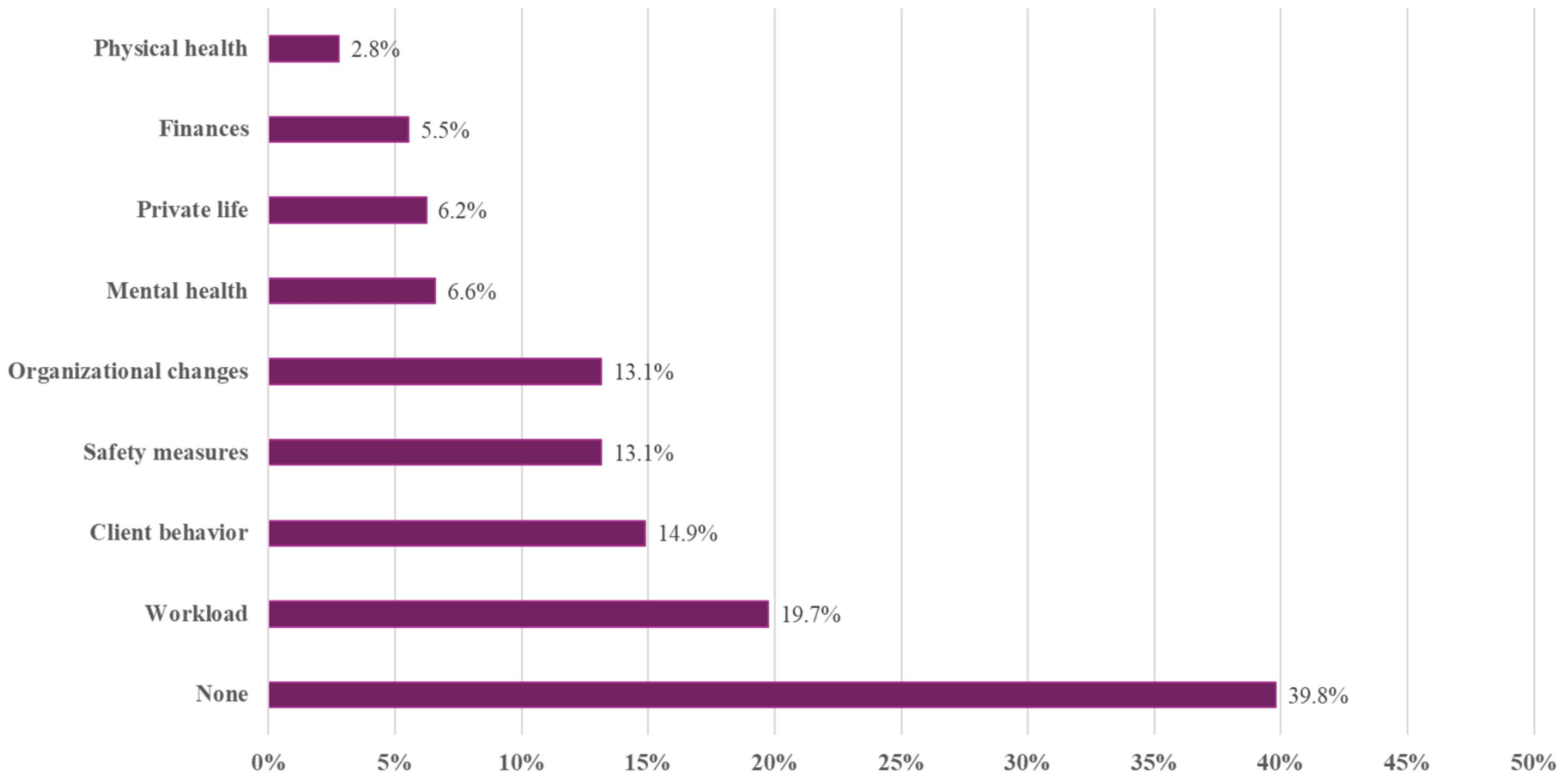
Percentage of respondents who reported one or more effects of the pandemic on the work as a veterinarian (main categories).

In the online survey of 440 veterinarians, 289 responded to the open-ended question, “What immediate or indirect impacts did the pandemic have on your work as a veterinarian?” The qualitative responses were content analyzed, yielding 9 main categories (Figure 1) and 19 subcategories (Table 3), with their respective percentages of the 289 responders.

**Table 3:** Category system that emerged from the question: “What direct or indirect impacts has the pandemic had on your work as a veterinarian?”

	*N*	%
None	115	39.8
Workload	57	19.7
Increase	45	15.6
Decrease	14	4.8
Client behavior	43	14.9
Financial constraints	15	5.2
Communication behavior	13	4.5
More demanding clients	12	4.2
Psychological and emotional changes	11	3.8
Owner-animal-dynamic	5	1.7
Safety measures	38	13.1
Mask usage	22	7.6
Less contact with clients	12	4.2
Issues related to vaccination	3	1.0
Organizational changes	38	13.1
Challenges	13	4.5
Staff-related issues	10	3.5
Shift to appointment-based system	8	2.8
Remote work/Home office	5	1.7
Delays in the delivery of medications	4	1.4
Mental health	19	6.6
Others	14	4.8
Fear of infection	6	2.1
Private life	18	6.2
Finances	16	5.5
Decrease	14	4.8
Increase	2	0.7
Physical health	8	2.8

Percentages relate to the sample of veterinarians who answered the open-ended question (*n* = 289). The percentages and total numbers of the main categories may differ from the sum of the percentages and numbers in the subcategories, as it might be that a veterinarian reported effects in several subcategories (e.g., financial constraints among clients and more demanding clients) within one main category (e.g., client behavior) and thus appears in each of these subcategories but is only counted once per main category.

As depicted in Figure 1, the most frequent mentions of veterinarians were, that the pandemic has not impacted their veterinary work (39.8%, *n* = 115). This category mainly comprised the single-worded statement “none,” or as Respondent Nr. 111 (R111) reported in more details: “None at all. Everything went quite normally.”

The second most-frequent statements were summarized as being related to workload (*n* = 57, 19.7%), with the majority (*n* = 45, 15.6%) reporting a rise in workload (Table 3). As an example, respondents stated: “Work more than doubled, no vacation for years” (R397), or “even more work than normal (we were the only practice still working) up to 70 patients divided between 2 vets/day” (R158). A lower proportion (4.8%, *n* = 14) reported a decrease in workload, such as “far fewer customers” (R410). Two veterinarians fell within both categories, as they experienced different changes in workload depending on the phase of the pandemic. As an example, R155 wrote: “Initially significant reduction in workload (first 40-h week), then absolute work peaks”.

A total of *n* = 43 (14.9%) veterinarians commented about client behavior. The most frequent statements related to financial constraints among clients (*n* = 15; 5.2%). Examples are R290: “Patient owners often do not have the financial means for treatments. As a result, it is often not possible to provide patients with the therapies they need.”, or R310 “… and we have to euthanize more animals where lives could perhaps be saved with more effort.” 13 veterinarians (4.5%) noted changes in communication (e.g., “Pet owners talk more about themselves,” R364). Further statements related to clients being more demanding (4.2%, *n* = 12). Changes in mental and emotional states of animal owners were reported by *n* = 11 (3.8%) veterinarians, such as “Customers are more “agitated”/more sensitive/receptive” (R121). Issues related to animal-owner-relationship were mentioned by 5 vets (1.7%). These statements related mainly to a surge in pet adoptions, such as R99 wrote: “Too many people have taken animals for the wrong reasons, which now overburdens them financially, timewise or otherwise.” Also reports about a rise in animal welfare issues were summarized in this subcategory.

A further main category related to safety measures, which were mentioned by *n* = 38 (13.1%) veterinarians. These encompassed mainly the usage of masks (*n* = 22, 7.6%) and decreased contact with clients (*n* = 12, 4.2%), whereby the latter was mainly commented in a positive way, such as: R77: “Less contact with patient owners was a relief,” or R158 “You have seen how easy it can be when the patient owner is not directly in the room.” A final subcategory summarized issues related to vaccination (*n* = 3, 1.0%). These statements were mixed ranging from side effects due to vaccination or public pressure related to vaccination to despair to delays in vaccination campaigns for veterinarians.

Several (*n* = 38; 13.1%) veterinarians commented about organizational issues. A total of *n* = 13 veterinarians (4.5%) faced various organizational challenges. This subcategory summarized unspecific statements such as “organisational problems,” “administrative expenses” (R432), but also statements related to additional burden related directly to the pandemic and associated containments efforts (e.g.: “changed workflows” R306, “No planning security. Legal requirements have been permanently changed.” R578). Another subcategory encompassed statements related to staff-related issues, which were named by *n* = 10 (3.5%) veterinarians. A total of 8 veterinarians (2.8%) reported about a shift to appointment-based system, which was mainly commented in a positive manner, such as “The introduction of an appointment practice has made my life much easier.” (R532). Further *n* = 5 (1.7%) veterinarians started working remotely. Finally, 4 veterinarians (1.4%) reported delays in the delivery of medications.

Issues related to mental health were named by *n* = 19 (6.6%) of the veterinarians. Statements from *n* = 14 (4.8%) veterinarians were generally brief, such as “mental burden” (R181) and could not be further differentiated. One subcategory encompassing statements from 6 veterinarians (2.1%) related to the fear of infection (*n* = 6, 2.1%).

Another main category related to aspects not related to professional activities *per se*. A total of *n* = 18 (6.2%) veterinarians reported that the pandemic impacted on their private life, with statements ranging from personal activities such as vacations, to issues related to homeschooling of their children.

Changes in the financial situation were reported by *n* = 16 (5.5%) veterinarians, with the majority (*n* = 14, 4.8%) reporting a worsening. The remaining two (0.7%) veterinarians experienced an improvement in finances, such as R104 stated: “Like many colleagues in the industry, the pandemic has made me a financial profiteer.”

The main category “physical health” encompassed statements from 8 veterinarians (2.8%), which related to negative health effects due to infection.

### 3.3 Mixed-methods results on the impact of the pandemic on the work of veterinarians

#### 3.3.1 Gender differences

The analysis revealed several significant gender differences in the impacts reported by veterinarians. Female veterinarians reported less frequently no impact of the pandemic on their work compared to their male counterparts (33.8% vs. 53.4%; *p* = 0.002). They reported changes in workload more frequently (22.4% vs. 12.5%; *p* = 0.041), i.e., a significant increase in workload (19.4% vs. 6.8%; *p* = 0.007). Female veterinarians also reported more often to encounter more demanding clients (6% vs. 0%; *p* = 0.021) and to experience financial impacts (7.0% vs. 1.1%; *p* = 0.044) than male veterinarians.

#### 3.3.2 Age differences

Older veterinarians (≥55 years) reported more frequently no impact of the pandemic on their work (40.8%) compared to their younger colleagues (17.1% in veterinarians aged 24–34, 26.7% in veterinarians aged 35–44, 22.2% in veterinarians aged 45–54; *p* = 0.001). Also, an association of age with reports on impacts on the client behavior were observed, with veterinarians aged between 45 and 54 reporting changes in client behavior more frequently (17.5%) compared to younger (7.2% in veterinarians aged 24–34, 6.7% in veterinarians aged 35–44) as well as older (6.1% in veterinarians aged 55 and older) veterinarians (*p* = 0.008).

#### 3.3.3 Impact related to treated animal species

Significant differences were observed based on the species of animals the veterinarians worked with. Those working with pets were less likely to report no impact (33.6% vs. 57.3%; *p* < 0.001) and more likely to report changes in workload (23.8% vs. 8%; *p* = 0.003) and a significant increase in workload (18.7% vs. 6.7%; *p* = 0.013). They also experienced more changes in the communication behavior of clients (6.1% vs. 0%; *p* = 0.024) and mental health issues (8.4% vs. 1.3%; *p* = 0.031). Conversely, veterinarians working with livestock reported more frequently no impact (49.5% vs. 33.7%; *p* = 0.007). They were also less likely to report an impact on workload (12.6% vs. 24.2%; *p* = 0.016) and an increase in workload (9.9% vs. 19.1%; *p* = 0.036). They were also less likely to report about more demanding clients (0.9% vs. 6.2%; *p* = 0.033), financial impacts (0.9% vs. 8.4%; *p* = 0.006), and decreased finances (0.9% vs. 7.3%; *p* = 0.012). However, they were more likely to report about delays in the delivery of medications (3.6% vs. 0%; *p* = 0.021). Those working with exotic animals reported less frequently no impact due to the pandemic (25.0% vs. 42.4%; *p* = 0.029), while they reported more organizational changes (27.3% vs. 10.6%; *p* = 0.003), organizational challenges (11.4% vs. 3.2%; *p* = 0.017), and a higher fear of infection (6.8% vs. 1.2%; *p* = 0.047).

#### 3.3.4 Self-employment status

Veterinarians who were self-employed, reported significant differences in several areas. They were more likely to report issues with communication behavior (6.5% vs. 1.0%; *p* = 0.036) and were less likely to mention safety measures (9.7% vs. 19.2%; *p* = 0.022). They also reported less impacts on reduced contact with clients (2.2% vs. 7.7%; *p* = 0.032) and were less likely to work remotely or from home (0% vs. 4.8%; *p* = 0.006). Financial aspects were less frequent among self-employed veterinarians (3.2% vs. 8.7%; *p* = 0.047), and they reported fewer negative effects due to infection (1.1% vs. 5.8%; *p* = 0.027).

#### 3.3.5 Curative practice

Veterinarians involved in curative practice were significantly less likely to report that the pandemic increased their remote work compared to those working in other areas of the veterinary profession (0.4% vs. 10.8%; *p* = 0.001).

### 3.4 Impact of the pandemic on the study of veterinary medicine

The COVID-19 pandemic has had varied impacts on veterinary medicine students, as captured in responses from 272 students. The analysis revealed 9 main categories and 16 subcategories, which are described in Table 4.

**Table 4:** Category system that emerged from the question: “What direct or indirect impacts has the pandemic had on your veterinary medicine studies?”

	*N*	%
Online/hybrid-learning	122	44.9
Negative	63	23.2
Positive	43	15.8
Neutral	20	7.4
Learning conditions	95	34.9
Decreased learning outcome	67	24.6
Less practical experience	42	15.4
Less concentration or motivation	19	7.0
Social distancing	94	34.6
Less social contacts	88	32.4
Impaired student life	7	2.6
Higher flexibility/less commuting	29	10.7
Mental health	29	10.7
Loneliness	13	4.8
Stress	12	4.4
(Social) anxiety	4	1.5
Depression	2	0.7
Sleep problems	1	0.4
None	22	8.1
Safety measures	15	5.5
Mask usage	11	4.0
Issues related to vaccination	3	1.1
Other measures	3	1.1
Organizational changes	13	4.8
Physical health	3	1.1

Percentages relate to the sample of veterinary students who answered the open-ended question (*n* = 272). The percentages and total numbers of the main categories may differ from the sum of the percentages and numbers in the subcategories, as it might be that a student reported effects in several subcategories (e.g., loneliness and depression) within one main category (e.g., mental health) and thus appears in each of these subcategories but is only counted once per main category.

**Figure 2:**
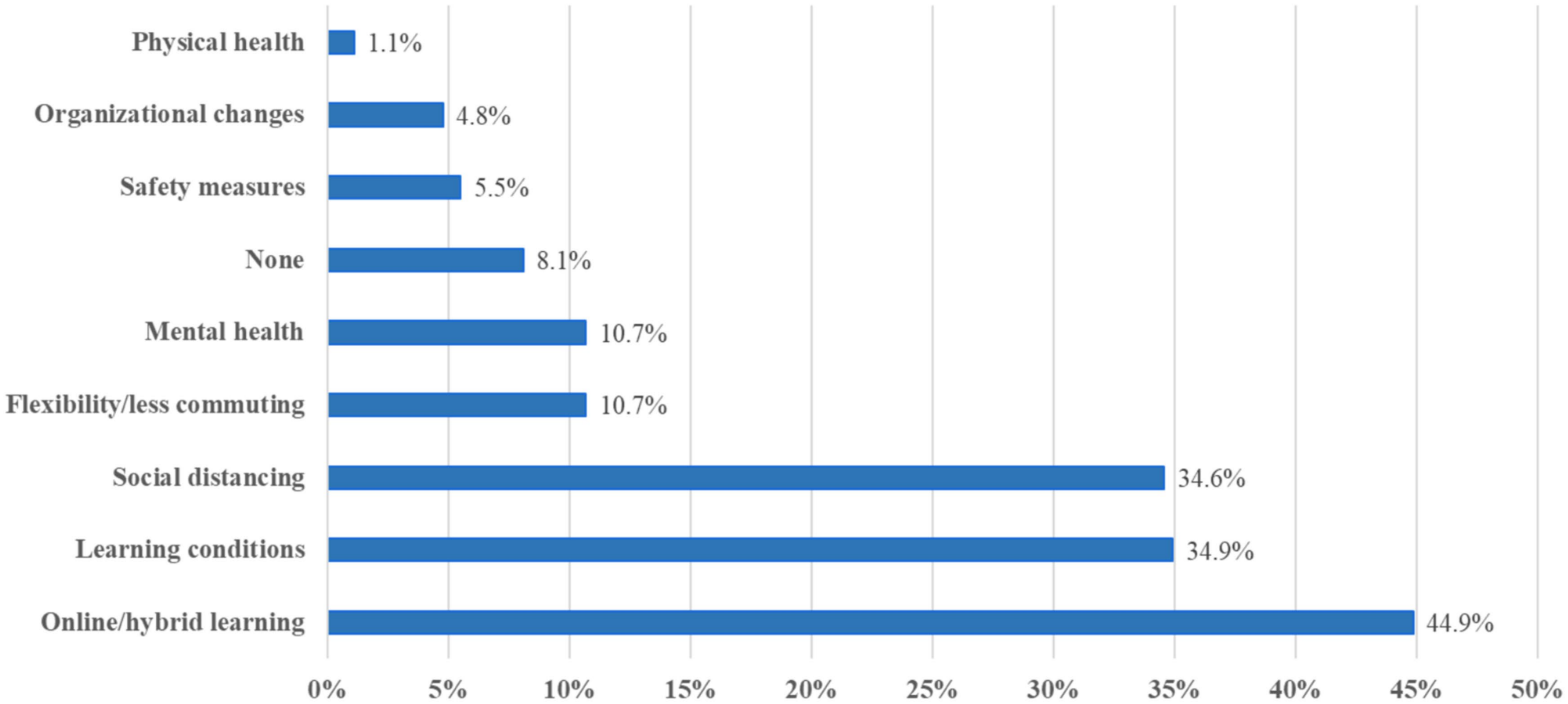
Percentage of respondents who reported one or more effects of the pandemic on the study of veterinary medicine.

The shift to online and hybrid learning was the most frequently mentioned impact, mentioned by *n* = 122 (44.9%) of the students (Figure 2; Table 4). Many students (*n* = 63, 23.2%) found this transition challenging, citing negative issues. Some students (*n* = 20, 7.4%) did neither comment positively nor negatively about online learning. They just mentioned statements such as “online learning” in the free text field without further comments. Conversely, several (*n* = 43, 15.8%) students mentioned positive aspects of online learning.

A second main category comprising statements from *n* = 95 (34.9%) students related to various effects of the pandemic on students’ learning conditions. A substantial number of students (*n* = 67, 24.6%) reported a decline in their learning outcomes, frequently attributing it to the lack of in-person classes. The reduction in hands-on practice was a significant concern for *n* = 42 (15.4%) students, impacting their preparedness for clinical work. R141 stated for instance “Many exercises were shortened or held in a different form; I sometimes feel less prepared for my professional life than I see with colleagues from the higher semester who have studied longer without COVID.” Some students (*n* = 19, 7.0%) also noted difficulties in maintaining concentration and motivation, which was often related to the remote learning environment. R265 wrote: “In online classes, I could hardly concentrate and missed a lot of material or did not understand and learn it well enough. As a result, I now feel hopelessly far behind in my studies compared to the requirements in the clinical rotations and at KlippVet.”

Social distancing measures were a further main category summarizing statements from *n* = 94 (34.6%) students. The most significant impact was the reduction in social interactions (*n* = 88, 32.4%), which many students found isolating. A small subcategory (*n* = 7, 2.6%) encompassed adverse effects on overall student life experience, with fewer opportunities for engagement in campus activities.

On the positive side, some students (*n* = 29, 10.7%) enjoyed greater flexibility and the elimination of commuting time, which allowed for a better balance between studies and personal life.

Mental health was another main area, mentioned by *n* = 29 (10.7%) students. Some students experienced feelings of loneliness (*n* = 13, 4.8%), such as R235: “I did not get to know anyone at the start of my studies and suffered extremely from loneliness. I was unable to cope with all the challenges at the start of my studies on my own.” Some students (*n* = 12, 4.4%) experienced changes in stress levels, mainly increased stress due to the uncertainty and changes brought by the pandemic. Social anxiety was noted by some students (*n* = 4, 1.5%), which was exacerbated by the isolation. Two (0.7%) students reported experiencing depression and one (0.4%) student mentioned sleep disturbances because of the pandemic’s impact.

A small group of students (*n* = 22, 8.1%) reported that the pandemic had no significant impact on their studies.

The implementation of safety measures was mentioned by *n* = 15 (5.5%) students. Wearing masks was the most mentioned measure (*n* = 11, 4.0%), which some found uncomfortable or disruptive. A few students (*n* = 3, 1.1%) mentioned concerns or issues related to vaccinations. Other safety measures such as COVID tests were also noted by *n* = 3 (1.1%) students.

Some students (*n* = 13, 4.8%) mentioned changes in the organization of their studies or institutions, affecting their academic routines.

A few students (*n* = 3, 1.1%) reported negative health effects due to COVID-19 infection.

### 3.5 Mixed-methods results on the impact of the pandemic on the study of veterinary medicine

#### 3.5.1 Country of origin

The analysis revealed only one significant difference in the impact of the COVID-19 pandemic on veterinary medicine students based on their country of origin. Specifically, a higher percentage of students from Austria (13.5%) reported enjoying higher flexibility and less commuting compared to students from other countries (3.8%; *p* = 0.017).

#### 3.5.2 Gender

Gender-based differences were observed only in the effects of the pandemic on students’ concentration and motivation. Female students reported significantly less effects on reduced concentration or motivation (5.1%) compared to male students (20.0%; *p* = 0.001).

#### 3.5.3 Study phase

The impact of the pandemic varied significantly across different study phases. Negative experiences with online learning were reported by 14.1% students in the first study phase, by 26.6% in the second phase and by 31.3% in the third phase (*p* = 0.022). The proportion of students reporting neutrally about online learning decreased from 12.1% in the first study phase to 6.4% in the second study phase, and 1.6% in the third study phase (*p* = 0.037). Positive experiences with online learning were reported by 30.3% of first-phase students, 7.3% of second-phase students, and 7.8% of third-phase students (*p* < 0.001).

Learning conditions showed significant variation as well, with 7.1% of first-phase students, 47.7% of second-phase students, and 56.3% of third-phase students reporting changes (*p* < 0.001). Decreased learning outcomes were reported by 6.1% of first-phase students, 30.3% of second-phase students, and 43.8% of third-phase students (*p* < 0.001). Less practical experience was noted by 1.0% of first-phase students, 22.0% of second-phase students, and 26.6% of third-phase students (*p* < 0.001). Less concentration or motivation was reported by 1.0% of first-phase students, 10.1% of second-phase students, and 10.9% of third-phase students (*p* = 0.014).

In terms of impaired student life, 1.0% of first-phase students, 0.9% of second-phase students, and 7.8% of third-phase students reported this issue (*p* = 0.010).

Higher flexibility and less commuting were appreciated by 16.2% of first-phase students, 10.1% of second-phase students, and 3.1% of third-phase students (*p* = 0.030).

No impact due to the pandemic was reported by 14.1% of first-phase students, 5.5% of second-phase students, and 3.1% of third-phase students (*p* = 0.019).

Safety measures affected 10.1% of first-phase students, 2.8% of second-phase students, and 3.1% of third-phase students (*p* = 0.043). Specifically, mask usage was reported by 10.1% of first-phase students, 0.9% of second-phase students, and none of the third-phase students (*p* = 0.001).

Organizational changes were noted by 1.0% of first-phase students, 3.7% of second-phase students, and 12.5% of third-phase students (*p* = 0.003).

## 4 Discussion

This study invited all Austrian veterinarians and veterinary students to share their experiences of the pandemic’s impact on the veterinary profession and education. Analyses of free-text answers revealed that the pandemic has profoundly affected veterinary medicine students and practitioners in multiple ways, with students seeming to be more affected than practitioners. While practitioners mentioned most frequently that they did not experience any effect of the pandemic, this was barley stated by the students. While practicing vets were mainly affected by changes in workload, the pandemic brought huge changes to the regular student life through online learning, social distancing, and less practical training. The specific experiences reported by each group are discussed in more detail in the following.

### 4.1 Impact on veterinarians

Veterinarians continued their work as essential services throughout the pandemic but faced increased workload, changes in client behavior, need to implement safety measures, and organizational changes. The high percentage of veterinarians (39.8%) reporting no pandemic-related impact on their professional activities suggests that veterinarians experienced relatively stable work conditions during the pandemic compared to the general public. This stability likely contributed to a less pronounced detrimental effect of the pandemic on their mental health (23), as supported by previous findings that individuals with low income (38) or those unemployed (39) in the Austrian general population faced higher odds of impaired mental health during the pandemic.

Despite this stability, the increased workload and associated stress have affected veterinarians, especially those working with pets, while those working with livestock experienced less impacts of the pandemic. The survey revealed that 19.7% of veterinarians reported changes in workload, with the majority experiencing an increase. This aligns with international trends observed among veterinarians. For instance, the Federation of Veterinarians of Europe (FVE) reported that 56% of European veterinarians experienced a rise in workload during the pandemic, with variations among countries (40). Austrian veterinarians were slightly below the average (47%); however, this is still considerably higher compared to the proportions of veterinarians reporting a rise in workload in the current study (15.6%). This discrepancy might be due to different phrasing and context of the survey question (pre-defined closed question in the FVE survey vs. free-text question on the impact of the pandemic on the work as a veterinarian in the study at hand).

Positive changes were also noted, such as a shift to appointment-based organization systems and reduced direct client interactions, which some veterinarians found beneficial. These organizational changes may have contributed to more efficient workflows and reduced stress levels.

Client behavior changes were another significant impact, with veterinarians noting financial constraints among clients, leading to challenges in providing necessary treatments. Additionally, some clients became more demanding or emotionally affected, reflecting broader societal stressors during the pandemic. This is in line with the FVE survey, where 33% of the participating veterinarians reported increased experience of bullying/harassment/conflicts with clients (40).

Safety measures, such as mask-wearing and social distancing, as well as delays in the delivery of medications were mentioned by a significant proportion of veterinarians, highlighting the additional layer of complexity in maintaining safe practice environments while managing increased demand.

Only 1.7% of veterinarians in our survey reported working remotely, and there were no specific mentions of telemedicine in the survey responses, which aligns with the findings, that almost all of these mentions came from veterinarians not working in the curative field. This contrasts with the FVE survey, where 25% of Austrian veterinarians reported an increased use of telemedicine since the pandemic took hold (40). Overall, it seems that online consultations gained more prominence in other countries, i.e., the UK, where almost half (46%) of the veterinarians reported an increase in the FVE survey (40). The limited adoption of remote work and telemedicine in Austria may reflect different regional practices or the unique nature of veterinary work that often requires hands-on examination and treatment of animals. As summarized recently, telemedicine is most effective for assessing whether an animal requires in-person visit and for follow-up consultations on existing cases but is insufficient for most diagnoses and treatments (41).

Financial impacts were noted by 5.5% of veterinarians, with the majority experiencing a decrease in income. This is consistent with broader economic challenges faced during the pandemic (27).

Additionally, 6.6% reported mental health issues, indicating that the increased workload and stress did take a toll on some veterinarians.

In summary, while a significant proportion of veterinarians reported no impact from the pandemic, a considerable number faced increased workload, changes in client behavior, and the need to implement new safety and organizational measures. These experiences of veterinarians mirrored many of these international trends. As essential workers, veterinarians continued to provide care for animals, but they had to navigate the complexities of increased demand and the implementation of safety measures such as mask-wearing and social distancing within clinics (42, 43). This period saw a surge in pet adoptions, further increasing the demand for veterinary services (9). Consequently, veterinarians had to manage higher workload, altered client interactions, and potential shortages of supplies due to disruptions in global supply chains (43–45).

### 4.2 Impact on veterinary students

Veterinary students experienced significant disruptions due to the COVID-19 pandemic, primarily stemming from the abrupt shift to online learning and the resultant loss of hands-on training and practical experiences. The survey responses highlight these challenges, with 44.9% of students noting the shift to online or hybrid learning as a major impact. The lack of in-person interaction and practical experience was particularly concerning for students, as veterinary education strongly relies on hands-on learning, from anatomy labs to clinical rotations.

The transition to online and hybrid learning formats was a mixed experience for veterinary students. While some students (15.8%) appreciated the benefits of online learning, a substantial proportion (23.2%) found it challenging. They reported reduced interaction with peers, impaired learning outcomes, and a lack of hands-on experience, essential for their training. The absence of practical exercises and clinical rotations left many students feeling underprepared for their future professional roles. Similarly, a previous study reported that while students appreciated the time management benefits and certain interactive elements of online learning, they were concerned about the lack of face-to-face interactions and technical issues, suggesting a need for strategies to enhance peer interaction and student-centered learning in online platforms (14).

The impact on learning conditions was significant, with 34.9% of students reporting a decline in their learning outcomes. The remote learning environment also posed difficulties in maintaining concentration and motivation, highlighting the struggle many students faced in adapting to the new learning modalities. This is in line with previous studies reporting that online learning formats in the study of veterinary medicine cannot fully replace real in-person settings (14, 21, 46, 47).

Social distancing measures also had a profound effect, with 34.6% of students reporting a reduction in social interactions, leading to feelings of isolation and loneliness. The lack of social engagement and peer support, which are critical components of the educational experience, exacerbated the challenges of remote learning. Additionally, the isolation from peers combined with the stress of adapting to new learning modalities, likely had significant implications for students’ mental health. Our companion study reports higher level of anxiety and depressive symptoms in Austrian veterinarians compared to the general public (22). A total of 10.7% of students reported increased mental distress due to the uncertainty and changes brought by the pandemic. The isolation and new learning environment contributed to social anxiety, loneliness, and depression among some students. The mental health impact on veterinary students aligns with broader trends observed in the general student population, where increased stress and anxiety levels have been widely reported during the pandemic (48, 49).

Despite the challenges, some positive aspects emerged. About 10.7% of students appreciated the higher flexibility and less commuting time associated with online learning. This allowed for a better balance between studies and personal life, illustrating that some adaptations could be beneficial in the long run.

While students in their final study phase experienced mainly negative effects related to online-learning, those within the first study phase reported primarily positive effects. The negative impact on final-phase students is likely due to the critical need for hands-on practice and clinical rotations during this period of their education. Without these experiences, they felt significantly underprepared for entering the professional field. Additionally, having previously experienced university life and education without the constraints of a pandemic, may have made the adjustment more challenging for these higher-phase students compared to those who have only known pandemic conditions throughout their studies. On the other hand, first-phase students benefited more from the flexibility and reduced commuting time, which allowed them to focus more on theoretical aspects and adapt gradually to the demands of their curriculum. The mixed-methods results highlight the necessity of a balanced approach, incorporating both online and in-person elements to meet the diverse needs of students at different stages of their veterinary education.

The pandemic has undoubtedly impacted veterinary students, emphasizing the importance of hands-on experience in their education, and highlighting the resilience required to adapt to unprecedented changes. While the shift to online learning presented numerous challenges, it also offered some benefits, such as increased flexibility. Moving forward, it is crucial for educational institutions to address the gaps in practical training and support students’ mental health and well-being. Integrating some of the beneficial aspects of online learning with hands-on training could enhance veterinary education and better prepare students for their future professional roles.

### 4.3 Limitations

This study has several limitations that should be considered when interpreting the findings. Firstly, the cross-sectional nature of the survey represents a snapshot in time and does not capture potential changes over the course of the pandemic or its aftermath. Secondly, the reliance on self-reported measures introduces the possibility of response bias. Participants may have underreported or overreported their experiences, perceptions, and stressors related to the pandemic and its impact on their studies and professional lives. Additionally, the study’s focus on Austrian veterinary students and licensed veterinarians may restrict the generalizability of the findings to other geographic regions. Furthermore, despite efforts to capture a diverse sample, there may be inherent biases in the recruitment process, particularly regarding participants who voluntarily chose to respond to an online survey. This could affect the representativeness of the sample and potentially introduce selection bias. Moreover, the study was conducted at the end of the third year of the pandemic (winter 2022/2023), when restrictions were minimal. While this timing could influence the reporting of experiences, as the situation had largely returned to normalcy, it also allowed participants to reflect on the full scope of the pandemic. Having experienced both peak and later stages of the pandemic, participants were in a unique position to provide comprehensive insights into its long-term impacts. However, some veterinarians may have returned to their regular routines, potentially overlooking or forgetting the direct impacts experienced during the peak of the pandemic. Lastly, the use of open-ended questions may have introduced variability in response depth and clarity, making data analysis more complex. While these questions can provide valuable qualitative insights, they also increase the risk of inconsistent or incomplete answers. To minimize this effect in future surveys, more structured prompts could be provided. In the current study open-ended questions were limited to areas where qualitative data were essential to provide insights into subjective experiences. However, the categorization and interpretation of open-ended responses are subject to researcher bias. Efforts were made to ensure reliability through rigorous coding procedures and team discussions, yet the possibility of overlooking certain perspectives or nuances cannot be entirely eliminated.

### 4.4 Conclusion

In conclusion, this study provides insights into the profound influence of the COVID-19 pandemic on both veterinary education and professional practice in Austria. The findings underscore the diverse challenges faced by students, influenced significantly by the pandemic’s disruptive effects on educational activities. Veterinarians reported relatively lower impact compared to students, as they were allowed to continue their professional activities relatively uninterrupted due to their role as essential workers, experiencing fewer restrictions. Future research should continue to monitor these impacts longitudinally and the potential for integrating beneficial practices, such as appointment-based systems, into standard veterinary care.

## Data Availability

The raw data supporting the conclusions of this article will be made available by the authors, without undue reservation.
